# Academic Career Pathways in German Medicine: Current Structures and International Comparisons

**DOI:** 10.7150/ijms.118738

**Published:** 2026-01-01

**Authors:** Markus Strauss, Sarah Altenberger, Jan Peter Ehlers

**Affiliations:** 1 Department of Cardiology I—Coronary and Peripheral Vascular Disease, Heart Failure Medicine, University Hospital Muenster, Cardiol, 48149 Muenster, Germany.; 2 Department Didactics and Educational Research in Health Science, Faculty of Health, University of Witten/Herdecke, 58455 Witten, Germany.

**Keywords:** academic career, doctorate, habilitation, professorship, tenure track, MD/PhD, science reform, medicine

## Abstract

**Introduction:** Academic qualification processes in medicine are an essential component of excellent research and teaching. In Germany, these qualification paths are characterized by heterogeneity in the regulations both between the federal states and between the faculties. This lack of uniformity in Germany stands in contrast to internationally established models with standardized and transparent career paths. In view of growing international mobility and increasing global competition, the need to reform academic careers in medicine in Germany is increasingly coming into focus.

**Methods:** On the basis of a systematic analysis, higher education laws, doctoral, habilitation and appointment regulations of German medical faculties were examined for defined target criteria. This was followed by a literature search in Pubmed and Google Scholar databases as well as freely accessible documents on the qualification systems of other international universities.

**Results:** The evaluation reveals considerable differences in the requirements, regulations and transparency of academic qualification levels in Germany. There is a lack of standardization processes in German regulations, particularly in the area of habilitation and extraordinary professorship. In an international comparison, there are clear differences between countries both in the existence of qualification levels and the qualification path to the qualification level. However, many countries have structured and transparent qualification levels with underlying tenure-track models or performance-oriented criteria.

**Conclusion:** The results of the academic qualification structures in medicine show a need for reform of the German academic system in an international comparison. The introduction of transparent and structured career paths, based on systems already established in other countries (PhD model/tenure track system), would contribute to harmonization and the promotion of international comparability and mobility

## Introduction

Academic qualifications in medicine form the basis for scientific progress, highly qualified teaching and excellent patient care. The assessment of academic performance is internationally regarded as a key instrument for ensuring the highest quality standards. However, there are sometimes significant differences between institutional and national guidelines when it comes to the criteria for academic qualification processes 1. In Germany, academic qualification is traditionally characterized by many institutional regulations and requirements, which are often regulated on a faculty-specific basis. Despite the high academic quality, the lack of standardization in this area poses a challenge for transparency, career progression and international connectivity.

In an increasingly globalized and networked scientific landscape, international connectivity is of fundamental importance. International comparability and recognition of medical qualification paths are of central importance in this context. The promotion and safeguarding of medical-academic careers is a strategic instrument for ensuring the quality of university healthcare and research, in addition to the recognition of individual scientific achievements.

The international mobility of doctors and scientists has been increasing for years, not least due to global crises. In 2023 alone, 64 thousand migrant doctors have been identified to practice their profession in Germany 2. Other reasons include the search for better research conditions, differences in training and recognition of scientific qualifications as more attractive career prospects. Transnationally compatible career paths are necessary to ensure optimal mobility. While countries such as the USA, the UK and Switzerland have established standardized career models with clear evaluation mechanisms and tenure track systems, Germany is dominated by a large number of individual regulations, particularly in the area of habilitation and extraordinary professorship. This makes mutual recognition more difficult and creates barriers to the promotion of young academics.

In other countries, the introduction of structured academic programs has promoted the development of a transparent and predictable academic career path. The structuring processes in other countries have made it possible to derive predictors that are linked to a successful academic career path. In a Canadian study by Schabort et al. 3, this was demonstrable for the factors of female gender, fluency in English, better results in USMLE Step 2 and participation in a skills test program. In Germany, on the other hand, there are still considerable differences in requirements and assessment criteria with regard to career paths on the basis of higher education legislation at state level and the individual regulations of the medical faculties. It can be assumed that this discrepancy has a negative impact on international connectivity and the attractiveness of the location.

Based on this background, the aim of this study is to analyze academic qualification levels in medicine in Germany and to present them in an international country comparison. The international comparison aims to identify structural differences. The aim is to identify potential for reform and to highlight prospects for greater international comparability and mobility.

## Methods

This work is based on a structured document analysis of academic qualification structures in medical education across Germany. The analysis considered the higher education laws of all 16 federal states as well as the doctoral, habilitation, and extraordinary professorship regulations of all 40 German medical faculties with valid regulations frameworks, taking into account all regulations up to April 2025. Only regulations from medical universities in Germany that are currently recognized by the German Council of Sciences and Humanities (Wissenschaftsrat) and the responsible ministries were taken into account. The analysis followed a set of predefined criteria, including admission requirements, qualification standards, evaluation metrics, and structural conditions. To complement this national perspective, recent statistical data from the German Federal Statistical Office (destatis.de) were analyzed to contextualize the prevalence and relevance of academic qualification stages. For the international comparison, a targeted literature search was conducted in PubMed and Google Scholar, covering publications available up to April 2025. In addition, publicly accessible university regulations and official policy documents from selected countries (including the United States, the United Kingdom, Switzerland, and Austria) were reviewed. The goal was to identify structural features and policy models of academic career progression in medical education and to compare them systematically with the German system.

## Results

**Figure 1:**
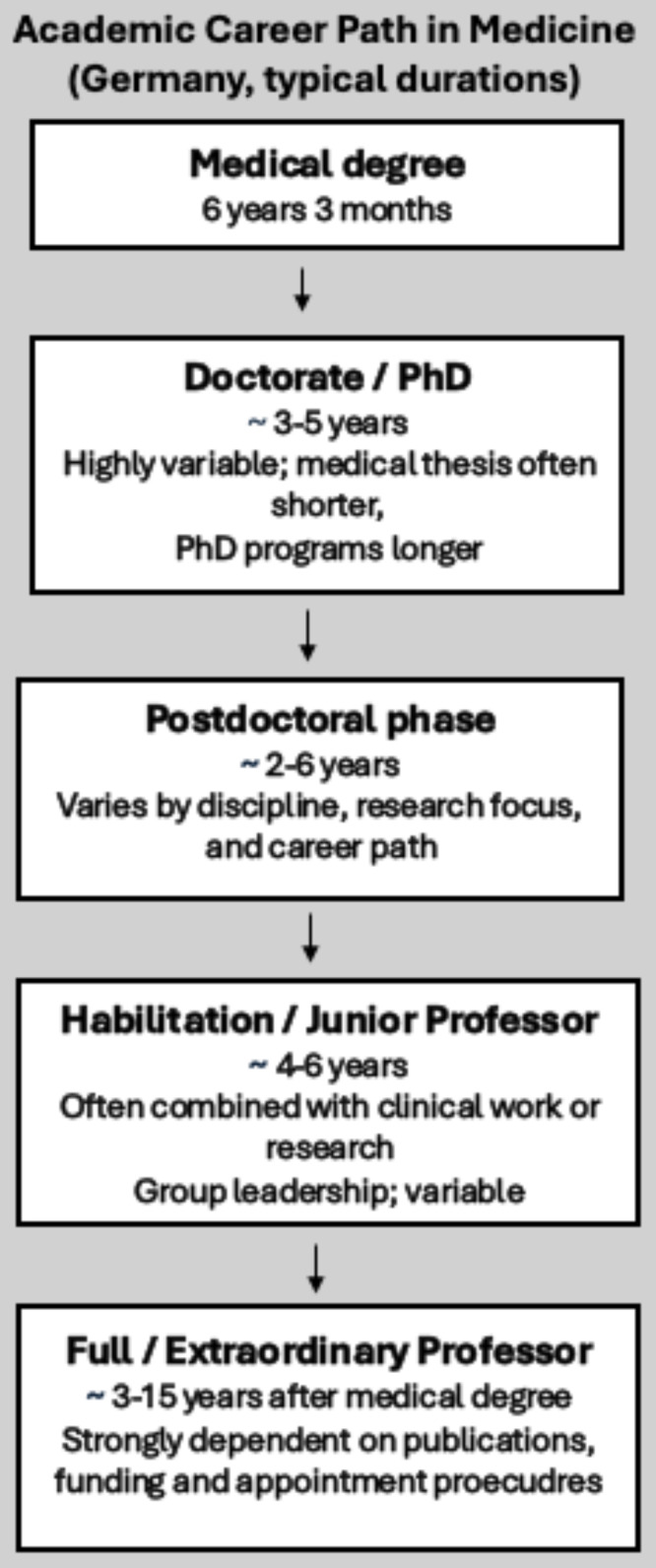
Stages of the academic career in Germany

The medical academic career in Germany comprises different stages (see Fig. 1). The framework for this is set by the higher education legislators of the state and the federal states. The following section provides an overview of these stages, which begin with the doctorate and end with the appointment of a professor. The doctorate or a Doctor of Philosophy program (PhD) is followed by the postdoctoral phase, which can lead to an extraordinary professorship or full professorship via habilitation or junior professorship.

### Higher education legislation in Germany

Academic career paths in medicine in Germany are largely shaped by higher education legislation. The basis is provided by Article 5 3 of the „Grundgesetz“ (basic law), which regulates freedom of science, research and teaching 4. The specific structure is determined by the respective state higher education laws (these vary between the federal states) and the respective university-specific or faculty-specific regulations. This situation leads to a heterogeneous legal situation that is difficult to compare, with sometimes considerable differences both between the federal states and the medical faculties. The lack of standardization leads to differences in terminology and structure as well as in the requirements and evaluation procedures of the doctoral and habilitation regulations and the regulations for extraoordinary professors. The doctorate and habilitation are anchored in higher education law and the respective state laws usually stipulate that universities must issue their own doctoral and habilitation regulations. The regulations set out basic rules; the specific requirements and procedures are not regulated in detail in the Higher Education Act, but in the respective faculty regulations. All 16 state higher education acts contain basic regulations on doctorates. The situation is similar for the habilitation. Here, for example, reference is made to paragraph 68 of the NRW Higher Education Act (Hochschulgesetz NRW) 5. Section 41 of the NRW Higher Education Act (Hochschulgesetz NRW) also sets out the requirements for obtaining an extraordinary professorship 5. However, not all higher education acts of the federal states stipulate criteria for the award of an extraordinary professorship. Compared to other scientific fields, the medical career is special in that there is a simultaneous obligation to clinical activity, research and teaching. This factor is not adequately reflected in higher education legislation. This leads to structural challenges in reconciling career requirements and real working conditions. The increasing establishment of structured programs such as PhD or Clinical Scientist initiatives have not yet been regulated by framework legislation and are currently written down individually for each university.

### Promotion

The first step in an academic career in Germany is the doctorate. This is an independent academic achievement by the doctoral candidate, which is intended to demonstrate their ability to work independently in an academic context. The doctorate consists of the preparation of a dissertation and the successful disputation stipulated in the rules. It can be of an experimental, clinical, statistical or theoretical nature. In addition to a monographic dissertation, a publication-based (cumulative) doctorate is now also possible at many faculties. This is characterized by the fact that the written dissertation work is usually provided by thematically related publications that have been published in peer-reviewed journals. In 2023, 86% (n= 45,838) of dissertations in the field of human medicine were written in monographic form and only 14% (n= 7,280) as part of a cumulative dissertation 6.

The requirements for the doctorate are defined by the faculties in their respective doctoral degree regulations. The doctoral thesis is supervised by a supervisor. The doctoral regulations stipulate who may take on this role. These are usually habilitated university lecturers or professors.

In Germany, practicing medicine requires a license to practice medicine from the state 7. This can be applied for after successfully completing a course of study lasting six years and three months. According to the regulations, the prerequisite for the admission of a doctoral candidate is often the successful completion of the first state examination. The doctorate can therefore already be started during the study of human medicine 8, 9. The conferral and use of the doctoral title through the awarding of the doctoral certificate is only possible after completion of the studies, i.e. the above-mentioned granting of the license to practice medicine.

According to figures from the Federal Statistical Office, there were a total of 204,945 doctoral students in Germany in 2023, with a median age of 30.4 years 6. Of the total of 204,945 doctorates, 48,413 (24%) of these doctorates were in human medicine. In a statistical comparison, human medicine is therefore still the subject with the most doctorates. A look at the gender distribution shows that in 2023, more women than men were working on a doctorate in human medicine 6.

### Doctor of Philosophy (PhD)

PhD programs are another factor in an academic scientific career. The Doctor of Philosophy or PhD is not subject-specific in its name and stands for the completion of a structured doctoral program. The focus of the structured program is an independent research project with dissertation and defense, which is completed within a specified period of full-time study. PhD programs originated in the USA, where MD/PhD programs were introduced in the middle of the last century 10, 11. This combines two courses of study and, over a period of seven to nine years, the medical degree Doctor of Medicine (MD) in the sense of a professional doctorate and the PhD in the sense of a research degree are obtained in parallel 12. Clinical scientist programs are also being introduced at German universities. However, the structure of the programs differs between the German medical faculties. One example of this is Ludwig Maximilian University, where a PhD can be obtained by completing a three-year full-time doctoral program after studying human medicine 13. At the University of Witten/Herdecke, there is a Clinical Scientist Program that provides access to experimental research through the development of a biomedical project parallel to specialist training and concludes with a PhD 14. As part of this program, an attempt is made to provide researchers with sufficient space both for clinical specialist training and for their scientific careers.

### Habilitation

Once proof of the ability to carry out independent academic work has been provided by the doctorate, the next qualification step follows: the habilitation. A successfully completed habilitation demonstrates the highest level of academic competence and the ability to teach independently. It is considered a key quality indicator for both research and teching performance 15-17. However, the habilitation does not exist as a qualification level in all countries. Table 1 presents a comparison of academic career stages across countries.

The framework conditions for awarding the habilitation are defined in the regulations of the medical faculties. A doctorate is a basic requirement. In addition, candidates must demonstrate along with continuous acdemic activity. While the doctorate focuses on a limited subject area, the habilitation proves broader knowledge in a more comprehensive field of research. In most cases, regulations require four years of clinical work or the attainment of a specialist title 15. A strong publication record is also essential. Other criteria include congress presentations, patents, and third-party funding. This period is known as the postdoctoral phase. Teaching performance is another requirement. Faculties define this in semester hours per week, with active teaching ranging from two to five years depending on the institution 15.

The habilitation process concludes with a written thesis- either monographic or cumulative-, a presentation to a scientific committee, and a public inaugural lecture. After successful completation, the habilitation certificate is issued. To obtain the "Venia legendi" (authorization to teach), a separate application must be submitted. Once granted, candidates may us the academic title Privatdozent (PD, Priv.-Doz.) and are obliged to teach regulary 16. The "Venia legendi" is the formal teaching license awarded by the university following successful habilitation and entitles the holder to teach independently at the university level. With this authoriziation, scholars may use the title Privatdozent, which denotes a person qualified to teach but without a professorial chair. Both the "Venia legendi" and the title Privatdozent are specific features of the German-speaking academic system and are mainly found in Germany, Austria, and Switzerland.

### Junior professorship

In 2002, the fifth amendment to the Higher Education Framework Act introduced the junior professorship 18. It represents an alternative to the habilitation, serves to further develop one's own research work and teaching experience and can be seen as preparation for a full professorship. The aim of introducing the junior professorship was to increase the attractiveness and competitiveness of academic careers internationally, to ensure more security and predictability for young academics and to prevent the exodus of German academics 18-20.

Junior professorships are usually limited to three or six years. At Witten/Herdecke University, the appointment is for three years 21. After this, an interim evaluation is required, during which a self-evaluation report must be written with a critical examination of one's own goals and successes already achieved. In addition, at least two reviews and the results of the teaching evaluations are included in the interim evaluation. If the interim evaluation is successful, the junior professorship is extended for a further three years 22. The junior professorship is possible either as part of a tenure track procedure or independently of it.

### Tenure track

Tenure track procedures have been established in the USA, but have also been increasingly introduced in Germany since the 2010s. In 2014, the German Science Council spoke out in favor of further developing junior professorships into tenure-track programs based on the American model in order to promote the attractiveness of academic careers in Germany 23. As part of a tenure track procedure, the appointment can be transferred to a full professorship after successful completion of a fixed-term appointment as a junior professor 24. The tenure track thus provides security and better planning for junior academics. Tenure track procedures are regulated in most universities' own regulations. The procedure at the University of Witten/Herdecke is described below as an example. It is stipulated that an evaluation procedure must be carried out no later than one year before the end of the fixed-term employment contract of the junior professorship 25. The applicant must have proven themselves in various areas, the evaluation criteria. The criteria cover the areas of research (national and international scientific cooperation, participation in conferences, publications, etc.), teaching (conception and implementation of courses, supervision of dissertations, Bachelor's and Master's theses, quality of teaching) and academic commitment (leadership/participation in internal university commissions and committees, management of study programs, expert activities, participation in foundations such as the German Research Foundation (DFG), Science Council, etc.) 25. In addition to the annual orientation meeting with the dean, mentoring is also planned in order to provide early assistance and support in the preparation of a self-report. In addition to the self-evaluation report, the procedure also provides for two external and one internal evaluation. The Tenure Track Commission is then responsible for preparing a report based on the results of the procedure, which are submitted to the dean. If the result is positive, the dean can finally submit a proposal to the faculty to transfer the appointment to a full professorship 25.

### Full professorship

The highest academic position in Germany is the full professorship, which includes the right to independent teaching and research. The requirements for an application are extensive. Experience in research and teaching must be demonstrated through a doctorate, habilitation or junior professorship. Appointment is made via a public advertisement and, after the application and assertion against possible competitors, leads to employment with a corresponding salary at the respective university. The employment relationship can be structured both in the original sense and in the form of a tenured position. Thus, a full professorship does not automatically mean a civil servant relationship. In order to become a civil servant, special requirements such as minimum age, state of health and commitment to loyalty to the constitution are necessary 26. The appointment process comprises various steps and begins with the advertisement of the position. This is followed by the application phase. In addition to their CV, applicants also submit their list of publications, research and teaching concepts, past third-party funding and proof of teaching experience/evaluations. An appointment committee, consisting of members of the faculty, student representatives, external reviewers and administrative staff, reviews the applications received. A ranking list of applicants is then drawn up, based on their performance in the interviews, appointment presentations and teaching presentations. The first-ranked candidate, also known as the call candidate, will be replaced by the second-ranked candidate if they do not take up the position. The third-placed candidate represents a further reserve in the event that both the first and second candidates do not take up the position. If the application is successful, the appointment to the professorship is accompanied by a teaching and research obligation. In addition to the areas of research and teaching, other tasks such as the management of study programs, faculties or institutes can be taken on. The basic salary of a professorship is based on three categories. The salary increases with each level. A W1 professorship corresponds to positions with fixed-term contracts, i.e. junior professorships. A W2 salary includes professors without a management function. In Germany, a W2 professorship is a mid-level academic position at a university, typically following the habilitation or an equivalent qualification. W2 professors are responsible for teaching, research, and participation in academic self-governance. A W2 salary includes professors without a managment function. The position can be tenured or fixed-term and offers more independence than a junior professorship (W1), though it is ranked below the full professorship (W3) in terms of salary. Finally, the W3 salary is the highest level and also includes the remuneration of a management position (e.g. chair holder). Performance bonuses can also be contractually agreed through negotiations. In addition to the basic salary, the acquisition of third-party funds or the recruitment of additional academic staff, for example, are then remunerated through performance bonuses.

A professorship may not be advertised if a suitable candidate is in a fixed-term employment contract and is suitable for the professorship offered 27. This is intended to counteract the exodus of qualified academic staff and take into account the university's internal promotion of young academics. It should be emphasized that the number of professors has increased steadily since 2014 and has risen from 3,789 in 2014 to 5,229 in 2023 in the human medicine/health sciences subject group 28. The number of female professors has almost doubled in the same period (from 726 in 2014 to 1508 in 2023). The proportion of female professors across all disciplines was 29% in 2023.

### Extraordinary Professor

The extraordinary professorship is one of the highest academic positions alongside the full professorship, but there are some differences 29. Although it is not possible to make a clear distinction between extraordinary professor and full professorships in this sense, as the title may also be used without the prefix 'extraordinary' may be used.

While an appointment procedure is necessary for a full professorship, this is not required for an extraordinary professorship. The requirements and conditions to be fulfilled by the applicant for appointment as an extraordinary professor are described in the regulations of the medical faculties and are based on the higher education framework laws of the respective federal states. One of the main requirements are the outstanding performance of the habilitated applicant in science, research and teaching. Most regulations stipulate a period of 2-6 years of teaching and research after the habilitation, during which the applicant must have proven themselves before a corresponding application can be submitted 30.

In principle, the extraordinary professorship demonstrates the smooth transitions between the individual career steps. At the University of Ulm, for example, the procedure for awarding an extraordinary professorship can already be carried out during the final evaluation of a junior professorship 31. A further indication that the different career stages should be taken into account is that almost 70% of extraordinary professorship regulations explicitly name a list placement for a full W2 or W3 professorship and this often results in the possibility of shortening the procedure for appointment as an extraordinary professor 30. Thus, a pure list placement can also be advantageous for the academic career path and increase the chances of an extraordinary professorship. In most cases, however, a previous junior professorship has no official influence on the award of an extraordinary professorship and is not mentioned in the regulations 30.

If the title of extraordinary professor is awarded, the award of the title does not automatically include an employment relationship, unlike in the case of a full professorship. Therefore, the main professional employment is usually outside the university. Nevertheless, holding the title is usually accompanied by an obligation to teach free of charge.

An international classification of the extraordinary professorship is only possible to a limited extent, as the extraordinary professorship is a unique feature that is only awarded at German universities.

## Discussion

### Need for reform in academic qualifications

Systematic career planning, continuous research activity, structured mentoring, institutional support structures and active scientific networking are considered key prerequisites for the long-term success of an academic career in medicine 32. A survey of German medical students by Sorg et al. 33 shows that there is still great interest in an academic career in medicine. Obtaining a title is seen as important for a professional career, but the career path from habilitation to a full professorship plays a subordinate role for the students surveyed.

Over the last 20 years, studies have repeatedly revealed major differences in the requirements for academic qualifications, although changes have also been recognizable over the last few years 34-36. For example, the updating of doctoral regulations in Germany in recent years has led to more detailed requirements in some places, but these have contributed little to standardization. Instead, the doctorate continues to present a picture of divergent regulations and requirements, some of which are imprecisely worded to allow scope for structuring, but do little to contribute to transparency, equal opportunities and the assurance of quality standards 37. The doctorate thus follows the example of other academic qualification processes in the sense of a habilitation or professorship, as there is still a great deal of divergence with regard to the regulations 15, 30. Accordingly, an analysis of the extraordinary professorship regulations carried out showed that, in addition to the differences in the individual assessment criteria, imprecise formulations also lead to individual scope for assessment and consideration on the part of the committees and reviewers 30. This scope for judgment works against the uniformity on the one hand and the transparency of the award procedures on the other. It can be assumed that the extraordinary professorship regulations at some faculties are understood more as guidelines, similar to what was assumed in the work by Nagelschmidt et al.^38^ for habilitation regulations. New, or at least not mentioned in earlier publications, are the points systems at four medical faculties. These point systems are not standardized, meaning that they are only comparable to a limited extent. The introduction of a nationwide uniform points system would be a practicable solution for standardizing the extraordinary professorship regulations.

As part of the revision of medical studies, efforts should also be made to standardize the doctoral system and to rethink the medical doctorate as such. Since the medical doctorate is an important instrument for securing scientific knowledge and recruiting young scientists, a standardization of the doctoral regulations should be tackled in a timely manner in order to improve transparency, comparability and fairness of achievements. One possibility, following the example of the Master Plan, is the appointment of a working group to define the inter-university framework on the basis of which the doctoral regulations could be oriented and revised. In addition, the discussion of a professional doctorate could also be discussed within this body. The German Science Council had already taken up this possibility in 2002 and reported on the possibility of introducing an "MD-like" title to replace the Dr. med. in the paper "Recommendations on doctoral training" 39. Potential candidates for a career in science and research should be offered PhD programs instead 39.

It is difficult to quantify the extent to which the demand for standardization of the extraordinary professorship regulations is pressing, as there is no data to date regarding the relevance of the extracurricular professorship. However, a survey conducted by Sorg et al. 40 among members of habilitation committees in 2016 makes it clear that the habilitation is still highly valued, even if the chances of a subsequent professorship are only considered to be average to low. As there have been no comparable surveys on the importance and evaluation of the extraordinary professorship to date, it can be assumed that the position is valued in the same way as the habilitation. The significant increase in requirements in the extraordinary professorship regulations also reflects the importance of the position, as the extraordinary professorship is ultimately a career factor that is associated with a certain reputational advantage, even if Alawi et al. 34 classifies the position of an extraordinary professor below that of a full professor (W2/W3). This gradation is justified by the fact that the extraordinary professorship is an unpaid teaching position. The appointment to a full professorship, on the other hand, is associated with a management function and personnel responsibility. A survey on the need to reform the regulations for extraordinary professorship, as is the case with the habilitation regulations, has not yet been carried out with regard to extraordinary professor 40. It can be assumed that the desire for nationwide uniform regulations, standardization and more transparency should also apply to the regulations for extraordinary professorship. After all, it is only possible to speak of fair conditions to a limited extent if the requirements for applicants are so divergent.

The number of doctoral and habilitation degrees is statistically recorded and evaluated in Germany. However, this factor is not available for extraordinary professorship and to date the Federal Statistical Office has not collected any data on this. An estimate can therefore only be made approximately on the basis of other data. The number of habilitations in the field of medicine has remained at a stable level in recent years 41. It can be assumed that the next step on the academic career ladder for habilitated academics is to aim for an extraordinary professorship or full professorship. On the one hand, it therefore seems possible to classify the number of extraordinary professors in relation to the number of habilitations. On the other hand, with an increasing number of professorships in the subject group of human medicine and health sciences, it can be assumed that the number of extraordinary professors has also increased over the last ten years 28. The question of the proportion of women allows further conclusions to be drawn, as the proportion has risen steadily in recent years. The proportion of female habilitation candidates rose from 18% in 2006 to 32% in 2019 and again to 37% in 2023 41, 42. The number of female professors has also risen and stood at 29% across all disciplines at the end of 2023 43. It can therefore be assumed that the proportion of female extraordinary professors has also increased. Nevertheless, the data shows that the higher the level of academic career, the lower the proportion of women 43.

Despite the clearly identified need for reform, the academic system in Germay has shown considerable resistance to standardization. Several factors may contribute to this. On a political level, the federal structure of Germany delegates higer education policy to the individual federal states (Bundesländer), resulting in decentralized decision-making and divergent regulations 44. This fragmentation limits the ability to enforce nationwide academic standards. Financial considerations also play a role. On a financial level, German universities vary considerably in funding stability and autonomy. The high reliance on project-based third-party funding and the reluctance to convert such positions into permanent roles makes the uniform implemenation of structured programs, such as tenure-track or standardized habilitation requirements, financially challenging 45. Finally, cultural factors are significant. The traditional prestige of the habilitation and the established hierarchical academic culture in Germany promote adherence to long-standing structures. Many faculties value flexibility and individual autonomy in academic appointments, which conflicts with efforts toward nationwide standardization 46, 47. These systemic characteristics help explain why reforms in German academic qualification processes are slow and often only partially implemented.

In summary, the academic regulations in Germany are in need of reform. Care should be taken to ensure that the classic compatibility of the three pillars of clinic, research and teaching at least remains and that reforms are undertaken to harmonize these in the future. A survey of doctors working in surgery has shown that, despite the prevailing economic pressure, there is a high level of motivation in this group of doctors for research and teaching 48. However, efforts need to be made to create structures that reward and promote commitment to research and teaching in a structured manner.

### Classification in the international context

#### International comparability of the medical doctorate

The international comparison of academic career paths is made difficult by the lack of standardized structures; Table 1 shows examples of the qualification levels in selected countries. As part of the Bologna Process in 1999 to standardize the higher education systems of European countries, adjustments have taken place, but there is still a large divergence with regard to the medical doctorate 49. Examples include Austria and the USA, which award the title of Dr. med. univ. or M.D. in the sense of a professional doctorate 50. This difference in degree designation is also reflected in dental education, where academic titles and qualification pathways differ significantly between Germany and the USA 51. This can be followed by separate doctoral studies or PhD studies, which lead to the title of Dr. med. scient. or PhD 11. PhD stands for Doctor of Philosophy and is not subject-specific in its naming. Obtaining the title requires a research achievement in the form of a dissertation and disputation. In addition, there is another option for students who want to dedicate themselves to research at an early stage through the so-called MD/PhD programs, which were introduced in the USA in the middle of the last century 10, 11. Within the framework of Medical Scientist Training Program (MSTP), two degree programs are combined so that the medical degree (M.D.) and the research degree (PhD) can be obtained in parallel over a period of seven to nine years 12. A systematic literature review by Straus et al. 52 showed that early research experience and publications, either during studies or as part of specialist training, promote interest in an academic career. Obtaining a doctorate or participating in a fellowship program has been shown to correlate strongly with a later academic career.

Although the USA has repeatedly faced the challenge of recruiting enough young academics in the past 53, 54, the MD/PhD programs are only accessible to a small proportion of students 10. On the one hand, they require a high level of willingness to work, meaning that they are primarily accessible to highly motivated students 55. Secondly, the programs are associated with high costs for the faculty and the National Institutes of Health due to tuition fee waivers or the awarding of scholarships 10. The MD/PhD programs therefore seem to make sense for students who want to prepare for an academic career early on and ensure that graduates have the opportunity to pursue better career paths 12, 56. Nevertheless, the number of graduates in these programs has declined 12. The main reasons for deciding against such programs appear to include the longer training period, the higher costs associated with a longer training period, the high competition for professorships and the compatibility of family and career 57. The extent to which this data is transferable to programs in Germany cannot be answered in detail. Based on the American PhD program, programs have also been established at European universities. These include German universities, for example Ludwig-Maximilians-Universität München (Germany), where a PhD can be obtained through a structured three-year full-time doctoral program following medical studies 13. The University of Witten/Herdecke (Germany) also offers a Clinical Scientist Program, which provides access to experimental research through the development of a biomedical project parallel to specialist training and can be completed with a PhD 14. Since October 2020, the Paracelsus Medical University in Salzburg (Austria) has also been offering a PhD course in addition to the human medicine degree, through which the internationally recognized title of PhD can be obtained within three years 58. In Switzerland, a study by the University of Geneva showed that following the introduction of MD/PhD programs, various obstacles exist, e.g. in the form of a double burden of clinical work and allocated research time, insufficient mentoring and funding, as well as difficulties in balancing family and career 59. One challenge here is certainly that, in contrast to the USA, PhD programs in Germany, Austria or Switzerland are completed after medical school. Although scholarships and funding programs are available, the start of a career and specialist training are delayed in the case of long-term clinical work. Nevertheless, as in the US, there appears to be an advantage for the further professional career of graduates, especially if they wish to pursue an academic career 12, 56, 59. A systematic literature review by Straus et al.^52^ showed that early research experience and scientific publications - whether during medical school or as part of specialist training - are associated with an increased interest in an academic career. A clear correlation was also found between obtaining a doctorate or participating in structured research programs (e.g. fellowship programs) and a later academic career. Against the background of these results, the targeted promotion of early academic qualification measures appears to be an effective approach to facilitate entry into an academic career and to promote its long-term continuation.

#### International comparability of the habilitation

The habilitation in the medical field is an academic qualification that plays a central role in academic careers, particularly in Central European countries. In Germany, Austria and Switzerland, the habilitation is still a formal qualification that entitles the holder to teach independently at a university and is often a prerequisite for a professorship. Figures from the Federal Statistical Office show that the habilitation continues to play a central role in the medical field in Germany. The number of habilitations increased by 4% in 2023 compared to the previous year and by far the most habilitations (60% of all procedures) continue to be carried out in the field of human medicine/health sciences 42. A survey of urologists revealed which factors are associated with the implementation of a successful habilitation. According to the survey, working time models, a research stay, a leave of absence and participation in funding programs or a mentoring program are independent predictors of a successful habilitation 60. However, a survey of habilitation candidates in Germany from 2016 makes it clear that a majority of respondents (86%) consider the habilitation to be in need of reform. The most important reform processes mentioned were the introduction of standardized national habilitation regulations, more transparency and less dependence on full professors 17.

In North America and the UK, there is no equivalent to the habilitation. In the UK, academic advancement is based on a step-by-step career progression from Lecturer to Reader and finally to Professor. These career steps are based on academic excellence and academic performance. In the USA and Canada, academic qualification is based on a postdoctoral system and cumulative academic achievements and publications. Appointment to a professorship is based on research success, teaching activities and the acquisition of third-party funding. Instead of the habilitation, there is a tenure-track professorship. As part of the tenure track, you have to prove that you are academically productive and have teaching skills within a certain period of time. A successful evaluation then leads to a permanent professorship. However, each higher education institution in the USA sets its own clear rules as to what candidates must achieve at the various career stages. The criteria therefore differ greatly between the individual universities in some cases. Overall, this leads to more flexibility and scope for assessment within the university system than in Germany. The situation is similar in the Scandinavian countries (Sweden, Norway and Denmark). Here too, there is no habilitation qualification level. Decisive consideration on the path to becoming a professor is given to research achievements, teaching activities and the acquisition of funding.

Overall, the habilitation is a traditional and established qualification in German-speaking countries with a unique selling point. It does not exist in other countries. On the one hand, the habilitation guarantees a scientifically sound education with a high level of teaching competence, but on the other hand it makes international comparability of academic performance more difficult and restricts the mobility of academics on international academic career paths. In the long term, the increasing spread of the tenure-track system in Germany could provide a remedy and bring about greater alignment with international standards.

#### International comparability of the professorial title

The academic positions at the upper end of the university career are structured similarly in many countries. In most countries, the appointment of full professor is the highest academic honor. The path to this position is characterized by high demands in the areas of research and teaching. While the basic qualification levels in medicine are similar to other subject areas, there are country-specific differences in the qualification process.

In many countries, the highest position within the academic hierarchy is associated with a lifelong appointment in the form of a professorship - internationally often referred to as a "chair". In Germany, appointment to a full professorship is based on a structured appointment procedure. A key requirement for this is either a completed habilitation or equivalent academic achievements in research and teaching.

In addition to full professorships, there are other academic positions in Germany with independent research and teaching. These include, in particular, W2 professorships, which generally do not have their own chair but are often associated with the management of a research group. Appointment to a W2 professorship also requires an appointment procedure. In an international comparison, this position corresponds most closely to Associate Professor in the USA or Senior Lecturer, Senior Researcher or Reader in the UK 61.

A special feature of the German higher education system is the titel of extraordinary professor, which is awarded exclusively by German universities and is difficult to compare internationally. In order to enable a classification, an exemplary comparison with the academic career paths in the USA and Great Britain is useful.

In the USA, academic promotion is regulated by the tenure-track system. After generally seven years as an assistant professor, promotion to associate professor is possible. This requires excellent research performance, successful acquisition of third-party funding and outstanding performance in teaching and clinical work. The position of associate professor therefore has certain parallels to the extraordinary professor in Germany 34, 62.

In the UK, an academic career begins as a Lecturer, a position comparable to Assistant Professor in the USA and 'Privatdozent' in Germany. Lecturers are generally employed on a fixed-term basis and work in both teaching and research. After three to four years, they can be made permanent. In the next career stage, a promotion to Senior Lecturer or Reader can be sought. While the Senior Lecturer corresponds to the American Associate Professor, the Reader can be compared to a German full professorship without a chair (W2 professorship).

Over the past ten years, some British universities have replaced the title of Reader or Senior Lecturer with Associate Professor. This measure is intended to facilitate international recruitment in particular. However, the appointment is initially limited to five years and is only terminated after a successful evaluation. The highest academic position in the UK is full professor, which is comparable to a W3 professorship in Germany.

## Conclusion

In Germany, there is a need for reform in the area of academic qualification processes. While doctoral regulations in their current form offer little transparency and comparability , the inconsistent handling of the criteria and processes for further academic careers makes equal opportunities and quality assurance more difficult. International comparisons show that many countries have already implemented standardized models that promote academic excellence and at the same time enable a more flexible academic career.

An international comparison shows that academic career paths in many countries are characterized by high demands on research and teaching. While in Germany the habilitation continues to play a central role in the appointment to a professorship, the USA and the UK have a more flexible system that is more strongly oriented towards academic performance. The extraordinary professorship in particular is a specific feature of the German higher education system and has no direct international equivalent. The overall picture is heterogeneous, which makes it difficult to compare academic careers on a global level. Greater standardization of academic qualification paths in Germany is therefore desirable for the future. Only through targeted reforms can Germany strengthen its position in the international academic system and make academic careers more attractive.

## Figures and Tables

**Table 1:** International comparison of academic career paths in medicine

Country	Doctorate	Habilitation	Professorial Ranks	Remarks
**Europe**				
Austria	Dr. med. univ.	Yes	University (or Full) Professor,Privatdozent	Habilitation required for full professorship; titles regulated nationally
Bulgaria	PhD	Yes	Lecturer, Professor	Habilitation required, nationally regulated
Czech Republic	PhD	Yes	Docent, Profesor	Habilitation required, professorship awarded by President
Denmark	PhD	No	Lecturer, Professor	No habilitation, institutional evaluations
Finland	PhD	Optional	Yliopistonlehtori, Apulaisprofessori, Professori	Docent title optional, varies by institution
France	PhD	Yes (HDR)	Maître de conférences, Professeur des universités	HDR necessary for professorship
Germany	Dr. med./PhD	Yes	W2/W3 Professorship	Habilitation traditionally required; alternative paths like junior professorships exist
Hungary	PhD	Yes	Egyetemi Docens, Egyetemi Tanár	Habilitation needed, professorship by presidential appointment
Italy	PhD	Yes (ASN)	Professore Associato, Professore Ordinario	National Scientific Qualification (ASN) required
Netherlands	PhD	No	Universitair Docent, Universitair Hoofddocent, Hoogleraar	Structured path, tenure-track common
Norway	PhD	No	Førsteamanuensis, Professor	Merit-based progression, no habilitation
Poland	PhD	Yes	Adiunkt, Profesor Uczelni, Profesor	Habilitation (dr hab.) needed, title conferred by President
Romania	PhD	Yes	Conferențiar, Profesor	Habilitation required for PhD supervision and professorship
Spain	PhD	Yes	Profesor Titular, Catedrático	National accreditation (ANECA) required
Sweden	PhD	No	Biträdande Lektor, Lector, Professor	Tenure-track available, no habilitation
Switzerland	Dr.med./PhD	Optional	Assistant, Associate, Full Professor	Mix of German and Anglo-American systems; habilitation varies by institution
**United Kingdom**				
England	PhD	No	Lecturer, Senior Lecturer, Reader, Professor	No habilitation, career progression by performance
**United States of America**				
USA	MD/PhD	No	Assistant, Associate, Full Professor (Tenure Track)	Tenure-track system prevalent, no habilitation

## Abbreviations

ANECA: Agencia Nacional de Evaluación de la Calidad y Acreditación (Spanish National Agency for Quality Assessment and Accreditation)
APL: Extraordinary Professorship
ASN: Abilitazione Scientifica Nazionale (Italian National Scientific Qualification)
DFG: Deutsche Forschungsgemeinschaft (German Research Foundation)
Dr. med.: Doctor of Medicine (German medical doctorate)
Dr. rer. nat.: Doctor of Natural Sciences
HDR: Habilitation à Diriger des Recherches (French postdoctoral qualification for supervising research)
MD: Doctor of Medicine (U.S./international title)
MD/PhD: Combined Doctor of Medicine and Doctor of Philosophy program
MSTP: Medical Scientist Training Program
NRW: North Rhine-Westphalia
PhD: Doctor of Philosophy
UK: United Kingdom
USA: United States of America
W1: Junior Professorship salary level (Germany)
W2: Associate Professorship salary level (Germany)
W3: Full Professorship salary level (Germany)

## References

[1] Lim BH, D'Ippoliti C, Dominik M, Hernández-Mondragón AC, Vermeir K, Chong KK (2025). Regional and institutional trends in assessment for academic promotion. Nature.

[3] Schabort I, Esfahani MA, Couban R, Roberts NW, Heneghan C, Arora N (2024). Predictors for success and failure in international medical graduates: a systematic review of observational studies. BMC Med Educ.

[8] Jüttemann A, Richter F, Wagner C, Dewey M (2014). [Development of the situation of doctoral students in medicine: Results of two surveys at an interval of ten years (2001 and 2011)]. Deutsch Med Wochenschr.

[9] Weihrauch M, Weber A, Pabst R, Weltle D, Lehnert G (2000). [The medical dissertation. An assessment from the viewpoint of successful and unsuccessful candidates]. Med Klin (Munich).

[10] Brass LF (2018). Is an MD/PhD program right for me? Advice on becoming a physician-scientist. Mol Biol Cell.

[11] Brass LF, Fitzsimonds RM, Akabas MH (2022). Gaps between college and starting an MD-PhD program are adding years to physician-scientist training time. JCI Insight.

[12] Alamri Y (2016). The combined medical/PhD degree: a global survey of physician-scientist training programmes. Clinical Medicine.

[15] Strauss M, Ehlers J, Gerß J, Klotz L, Reinecke H, Leischik R (2020). [Status Quo - The requirements for medical habilitation in Germany]. Deutsch Med Wochenschr.

[16] Weineck SB, Koelblinger D, Kiesslich T (2015). [Medical habilitation in German-speaking countries: Quantitative assessment of content and elaboration of habilitation guidelines]. Der Chirurg; Zeitschrift fur alle Gebiete der operativen Medizen.

[17] Sorg H, Betzler C, Grieswald C, Schwab CG, Tilkorn DJ, Hauser J (2016). [Postdoctoral lecturer thesis in medicine: academic competence or career booster?]. Chirurg.

[18] Breithaupt H (2005). A focus on the individual. Germany has begun to reform its university system to make it more attractive to both foreign and German scientists and students. EMBO Rep.

[19] Burkhardt A, Nickel S, Berndt S, Püttmann V, Felix A (2016). Die Juniorprofessur -vergleichende Analyse neuer und traditioneller Karrierewege im deutschen Wissenschaftssystem. Beiträge zur Hochschulforschung.

[20] Pantenburg B, Kitze K, Luppa M, König HH, Riedel-Heller SG (2018). Physician emigration from Germany: insights from a survey in Saxony, Germany. BMC Health Serv Res.

[21] Herdecke UW APL Ordnung Universität Witten/ Herdecke. 2016.

[29] Sorg H, Betzler C, Grieswald C, Schwab C, Tilkorn D, Hauser J (2016). Die medizinische Habilitation: akademische Befähigung oder Karriereinstrument?. Der Chirurg.

[30] Altenberger S, Leischik R, Vollenberg R, Jehn U, Reinecke H, Ehlers JP (2021). Requirements for Becoming an Adjunct Professor in Medicine: A Comparative Analysis of the Regulations of German Medical Faculties. Int J Environ Res Public Health.

[32] Howard-Anderson JR, Gewin L, Rockey DC, Krousel-Wood M, Roman J (2024). Strategies for developing a successful career in academic medicine. Am J Med Sci.

[33] Sorg H, Ehlers JP, Zupanic M, Salehi I, C GGS (2023). [How important is an academic career in medicine today? A survey among medical students in Germany: Results of study arm XIII of the KARiMED study]. Z Evid Fortbild Qual Gesundhwes.

[34] Alawi SA, Luketina R, Krezdorn N, Busch LF, Limbourg A, Branski L (2019). How to become a medical professor - a comparative analysis of academic requirements in Germany and the United States. Innov Surg Sci.

[35] Pabst R, Strate J (2000). [Major differences in procedure in receiving the academic title "ausserplanmaessiger Professor". Criteria and evaluation of research and teaching at German medical schools]. Der Chirurg; Zeitschrift fur alle Gebiete der operativen Medizen.

[36] Sorg H, Knobloch K (2012). Quantitative evaluation of the requirements for the promotion as associate professor at German medical faculties. GMS Zeitschrift für Medizinische Ausbildung.

[37] Altenberger S, Leischik R, Vollenberg R, Ehlers JP, Strauss M (2024). A comparative analysis of the doctoral regulations at the medical faculties in Germany. International Journal of Medical Sciences.

[38] Nagelschmidt M, Bergdolt K, Troidl H (1998). [Evaluation of qualification regulations for medical faculties of German universities and recommendations for standardization]. Chirurg.

[40] Sorg H, Kramer R, Grieswald C, Schwab CG, Thonnes S, Reinke JM (2016). [Assessment of the significance and the requirements of medical postdoctoral lecture qualifications in Germany by the assessment committees]. Zeitschrift fur Evidenz, Fortbildung und Qualitat im Gesundheitswesen.

[43] Bundesamt S (2024). Pressemitteilung Nr. 459 2024 [cited.

[44] Berghaeuser H, Hoelscher M (2020). Reinventing the third mission of higher education in Germany: political frameworks and universities' reactions. Tertiary Education and Management.

[45] Kuhnt M, Müßig P, Reitz T (2024). There are alternatives. Models for sustainable employment structures in the German system of higher education. Front Res Metr Anal.

[46] Hamann J (2019). The making of professors: Assessment and recognition in academic recruitment. Social Studies of Science.

[47] Wilkesmann U, Wagner O (2024). Theoretical and empirical approach to how a professorship is organized in the German higher education system and how the organizational process works. Higher Education.

[48] Roeth AA, Jauch D, Buhr HJ, Klinger C, Sommer N, Wachter N (2023). Zukunft der Universitätsmedizin: Welchen Stellenwert haben Forschung und Lehre noch?-Eine Bestandsaufnahme. Zentralblatt für Chirurgie-Zeitschrift für Allgemeine, Viszeral-, Thorax-und Gefäßchirurgie.

[51] Vargas N, Romanos GE (2022). Dental Academic Degrees in Germany Compared to the USA. Dent J (Basel).

[52] Straus SE, Straus C, Tzanetos K (2006). Career choice in academic medicine: systematic review. J Gen Intern Med.

[53] Milewicz DM, Lorenz RG, Dermody TS, Brass LF (2015). Rescuing the physician-scientist workforce: the time for action is now. The Journal of clinical investigation.

[54] Ley TJ, Rosenberg LE (2005). The physician-scientist career pipeline in 2005: build it, and they will come. Jama.

[55] Andriole DA, Whelan AJ, Jeffe DB (2008). Characteristics and career intentions of the emerging MD/PhD workforce. Jama.

[56] Brass LF, Akabas MH, Burnley LD, Engman DM, Wiley CA, Andersen OS (2010). Are MD-PhD programs meeting their goals? An analysis of career choices made by graduates of 24 MD-PhD programs. Academic medicine: journal of the Association of American Medical Colleges.

[57] Kersbergen CJ, Bowen CJ, Dykema AG, Koretzky MO, Tang O, Beach MC (2020). Student perceptions of MD-Ph. D. programs: a qualitative identification of barriers facing prospective MD-Ph. D. applicants. Teaching and learning in medicine.

[59] Dos Santos Rocha A, Combescure C, Negro F (2020). The MD-PhD program in Geneva: a 10-year analysis of graduate demographics and outcomes. BMC Med Educ.

[60] Welte M-N, Knipper S, Siech C, Greiser EM, Wiemer L, Müller K (2022). Frauenförderung in der Urologie am Beispiel der Habilitation. Die Urologie.

[61] Kreckel R (2016). Zur Lage des wissenschaftlichen Nachwuchses an Universitäten: Deutschland im Vergleich mit Frankreich, England, den USA und Österreich. Beiträge zur Hochschulforschung.

[62] Kaplan K (2010). Academia: The changing face of tenure. Nature.

